# Two-year analysis of *Clostridium difficile* ribotypes associated with increased severity

**DOI:** 10.1016/j.jhin.2019.06.003

**Published:** 2019-12

**Authors:** R. Herbert, J. Hatcher, E. Jauneikaite, M. Gharbi, S. d’Arc, N. Obaray, T. Rickards, M. Rebec, O. Blandy, R. Hope, A. Thomas, K. Bamford, A. Jepson, S. Sriskandan

**Affiliations:** a Imperial College Healthcare NHS Trust, London, UK; b NIHR Health Protection Research Unit in Healthcare Associated Infections and Antimicrobial Resistance, Imperial College London, London, UK; c National Infection Service, Public Health England, London, UK

**Keywords:** *Clostridium difficile*, Ribotype, Severe infection, C-reactive protein, tcdA, tcdB

## Abstract

**Background:**

Certain *Clostridium difficile* ribotypes have been associated with complex disease phenotypes including recurrence and increased severity, especially the well-described hypervirulent RT027. This study aimed to determine the pattern of ribotypes causing infection and the association, if any, with severity.

**Methods:**

All faecal samples submitted to a large diagnostic laboratory for *C. difficile* testing between 2011 and 2013 were subject to routine testing and culture. All *C. difficile* isolates were ribotyped, and associated clinical and demographic patient data were retrieved and linked to ribotyping data.

**Results:**

In total, 86 distinct ribotypes were identified from 705 isolates of *C. difficile*. RT002 and RT015 were the most prevalent (22.5%, *N*=159). Only five isolates (0.7%) were hypervirulent RT027. Ninety of 450 (20%) patients with clinical information available died within 30 days of *C. difficile* isolation. RT220, one of the 10 most common ribotypes, was associated with elevated median C-reactive protein and significantly increased 30-day all-cause mortality compared with RT002 and RT015, and with all other ribotypes found in the study.

**Conclusions:**

A wide range of *C. difficile* ribotypes were responsible for *C. difficile* infection presentations. Although *C. difficile*-associated mortality has reduced in recent years, expansion of lineages associated with increased severity could herald increases in future mortality. Enhanced surveillance for emerging lineages such as RT220 that are associated with more severe disease is required, with genomic approaches to dissect pathogenicity.

## Introduction

*Clostridium difficile* infections (CDI) remain a significant cause of mortality and morbidity [1]. In England, the infection rate for all reported cases of CDI is 40.8/100,000 bed-days, representing 14,139 infections per year [2]. CDI rates in the UK have decreased since 2007, likely due to a number of infection control and antibiotic stewardship interventions, and also, in part, to a decline in the epidemic ribotype RT027, which had been associated with poor clinical outcomes [1], [2], [3], [4]. Ribotypes such as RT018, RT027, RT056, RT078, RT176 and RT244 have all been reported to be associated with complicated disease outcomes, recurrences and increased severity [4], [5], [6], [7], [8], [9], [10], [11]. However, it is not yet known whether ribotype can predict the severity of infection *per se* [12], [13].

A two-year project was undertaken to implement routine ribotyping of all *C. difficile* isolates, and evaluate the impact of this surveillance on infection prevention strategy and management of outbreaks. As part of the study, linked, routinely collected clinical, demographic and microbiological data with ribotype results were analysed to determine if any particular ribotype was associated with an adverse outcome in patients.

## Methods

### Study population and routine microbiological identification of C. difficile episodes

Stool samples from five major West London acute hospitals and associated primary care centres were submitted to a single diagnostic laboratory, that serves a population of approximately 2 million people, for *C. difficile* testing. Between August 2011 and June 2013, all faecal samples from patients aged >2 years with diarrhoea that tested positive using routine tests for *C. difficile* were included in the study. Confirmation of a *C. difficile* episode required detection of glutamate dehydrogenase (GDH) and *C. difficile* toxin B gene by polymerase chain reaction (PCR) (Xpert *C. difficile* PCR, Cepheid Inc., Sunnyvale, CA, USA) in stool. Stool samples submitted for *C. difficile* testing that were received within 28 days of a previous positive result were not included in this study. Stool samples from confirmed *C. difficile* episodes obtained after April 2012 also underwent toxin enzyme immunoassay (EIA) testing (C.DIFF QUIK CHEK COMPLETE, Alere Inc., Waltham, MA, USA) following a change in UK guidelines [14] (Figure S1, see online supplementary material)

### Culture and characterization of C. difficile isolates

Each sample obtained from a *C. difficile* episode was cultured using the alcohol shock method, followed by culture on Brazier's *C. difficile* selective agar [15]. *C. difficile* isolates were identified by matrix-assisted laser desorption ionization-time of flight mass spectrometry (MALDI-ToF MS, Bruker Corp., Billerica, MA, USA) and stored as glycerol stocks at -80^o^C. Samples that failed to grow were recultured. If a sample failed to grow when recultured, or if there was inadequate sample to reculture, they were recorded as such and excluded from the final analysis. Confirmed *C. difficile* isolates were subsequently characterized using standard PCR ribotyping [16] (Figure S1, see online supplementary material). Isolates that could not be ribotyped were retyped by the Anaerobic Reference Laboratory, Cardiff. Antimicrobial resistance testing was not undertaken routinely.

### Clinical data and CDI episode classifications

*C. difficile* episodes were linked to routinely collected demographic, microbiological and clinical data, where available, via electronic databases prior to anonymization. Patient location at time of sampling, length of hospital admission, medical speciality (medicine/surgical/general practice or other, including paediatrics, obstetrics and gynaecology, emergency medicine and intensive care), International Classification of Diseases (ICD)-10 coding, toxin EIA production, C-reactive protein (CRP) (mg/L), leukocyte count (x10^9^/L), albumin (g/L), creatinine (μmol/L) and mortality data were collected. Patients with known renal disease were excluded from analyses where creatinine level was a variable. Biomarkers of severity, namely CRP, total leukocyte count, albumin and creatinine, measured within 48 h of *C. difficile* sampling (date of sample ± 24 h) were recorded. Mortality records were traced on 27^th^ September 2016 and were de-duplicated.

Individual isolates of *C. difficile* were considered to represent a separate episode when identified more than 28 days apart. Recurrence was defined as any additional *C. difficile* episode in the same patient beyond 28 days. In those patients with a recurrence, a relapse of infection was defined as re-isolation of the same ribotype more than 28 days after the original *C. difficile* episode [17]. Clinical samples testing positive for *C. difficile* that were submitted more than 72 h after admission to hospital were considered as hospital-onset. Samples submitted on admission to hospital or within 72 h of admission were considered as community-onset in line with national guidelines at that time [14]. Samples submitted by community doctors or from an outpatient clinic were also considered as community-onset. If the date of hospital admission was not known, no classification was attempted.

### Statistical analysis

The primary outcome measure was 30-day all-cause mortality from date of detection of GDH and positive toxin B PCR result. Secondary outcome measures were mean length of hospital stay in days (excluding patients that died within 30 days and/or patients with recurrent infection during same hospital admission) and biomarkers associated with disease severity [18], [19].

The primary exposure was *C. difficile* ribotype. Covariables considered were age and sex. Preliminary data analysis revealed that RT220 was associated with increased mortality compared with the other most frequently (>3% prevalence) isolated ribotypes, warranting further analysis (Figure S2, see online supplementary material).

The mortality rates of three groups were compared using Kaplan–Meier curve and log-rank (Mantel-Cox) test: RT220 was compared with the two most frequently isolated ribotypes in this study, RT002 and RT015, and with all other ribotypes isolated. In doing so, RT002/RT015 and the heterogeneous group of all other ribotypes (which included some rare ribotypes) acted as separate, large control groups. After ensuring the proportional hazard assumptions were met, a multi-variable Cox regression analysis was performed to assess associations between ribotype and all-cause mortality within 30 days. The model was adjusted for sex and age to account for potential sources of confounding.

One-way analysis of variance was used to compare the mean length of hospital stay between the three groups, and Kruskall–Wallis test and Mann–Whitney *U*-test were used to compare biomarkers between groups. Prevalence of ribotypes and proportions for categorical variables were compared using Chi-squared test. Additional variables, where available, compared between groups included toxin EIA, source of infection (hospital or community onset) and modified Elixhauser score (a method of categorizing comorbidities of patients based on ICD diagnostic codes) [20]. All *P*-values were based on a two-tailed test with *P*<0.05 considered to indicate significance. Data were recorded in Excel (Microsoft Corp., Redmond, WA, USA) and analysed using GraphPad Prism Version 7.0 (GraphPad Software, San Diego, CA, USA).

### Ethics

The collection and analysis of bacterial isolates and linked anonymized clinical data from patients was approved by the West London NHS Research Ethics Committee (Ref. 06/Q0406/20). Methods were in accordance with the specified approved protocol and regulations.

## Results

In total, 758 GDH and PCR toxin-B-positive *C. difficile* episodes were identified in the study period, from which 705 *C. difficile* isolates were ribotyped (Figure S1, see online supplementary material). The median age of all patients for whom a *C. difficile* ribotype was reported was 74 years (interquartile range 60–83 years).

**Figure 1:**
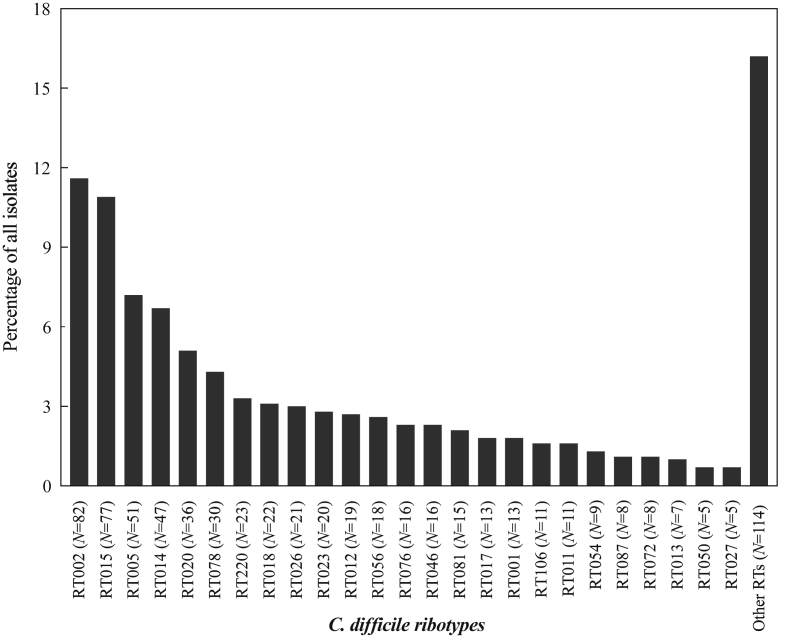
Frequency of *Clostridium difficile* episodes caused by each ribotype over a two-year surveillance period. In total, 705 isolates were assigned a ribotype. The 25 most common ribotypes (isolated in five or more episodes) are shown; the other 61 ribotypes were grouped together as ‘Other RTs’. Number of isolates per ribotype group are provided (*N*).

Eighty-six different ribotypes were identified (Figure 1). The 10 most common ribotypes were: RT002 (11.6%), RT015 (10.9%), RT005 (7.2%), RT014 (6.7%), RT020 (5.1%), RT078 (4.3%), RT220 (3.3%), RT018 (3.1%), RT026 (3.0%) and RT023 (2.8%). RT027 was isolated in only five patients (0.7%). Routine toxin EIA testing was introduced in April 2012, and 189 (43.2%) of 438 stool samples tested were toxin EIA positive.

Mortality records were available, where NHS number was recorded, for 463 of 705 (65.6%) episodes with ribotypes, representing 450 individual patients. Of the 450 patients with mortality data available, 90 (20%) died within 30 days of *C. difficile* sampling. No difference was detected in the prevalence of specific ribotypes or toxin positivity between patients for whom mortality data were available and those for whom mortality data were unavailable.

**Figure 2:**
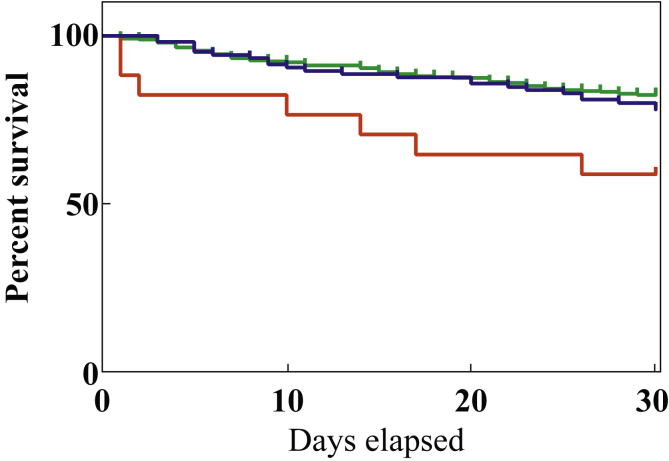
Kaplan–Meier survival plot showing 30-day all-cause mortality in patients with *Clostridium difficile*; patient population analysed by ribotype (*N*=number of episodes for which mortality/survival data were available). *P*=0.02 (log rank test). Red line, RT220 (*N*=17); blue line, RT002 and RT015 (*N*=105); green line, all other ribotypes (*N*=341).

Focusing on the most common ribotypes (with >3% prevalence) within the study population, survival analyses demonstrated a reduced 30-day survival for CDI episodes caused by RT220 (Figure S2, see online supplementary material), which was confirmed when compared with survival for episodes caused by the two dominant ribotypes, RT002 and RT015 (*P*=0.049), and all other ribotypes (*P*=0.006) (Figure 2). There were 23 episodes of RT220 in 21 patients; mortality data were available for 17 of 21 patients (and 17 of 23 episodes). All-cause mortality at 30 days was 41.2% (7/17) for RT220 compared with 21.9% (23/105) for RT002/RT015 (Table I). Where data were available, RT220 deaths occurred in older patients, diagnosed in both community and hospital settings by a range of clinical specialities (Table S1, see online supplementary material). From the available clinical data, female patients were affected by RT220 more frequently than males, but this difference was not significant (*P*=0.096). Half of the *C. difficile* episodes with ribotypes had hospital-onset CDI; RT220 episodes were no different to episodes with other ribotypes (Table I). Multi-variable Cox regression analysis using a model adjusted for sex and age showed a 2.46-fold increased risk of 30-day mortality in patients with RT220 compared with both control groups (Table II).

**Table I:** Characteristics of 705 *Clostridium difficile* episodes with ribotyped isolates

Ribotype	RT220 Total (%)	RT002 + RT015 Total (%)	All other ribotypes Total (%)	*P-*value^b^
Demographic data				
No. of *C. difficile* episodes with ribotyped isolates (*N*=705)	23 (3.3)	159 (22.6)	523 (74.2)	
Total no. of unique patients (*N*=672)	21 (91.3)	153 (96.2)	498 (95.2)	0.57
Age range of *C. difficile* episodes (years)				
<65	4 (17.4)	45 (28)	165 (31.5)	0.17
65–79	13 (56.5)	54 (33.9)	168 (32.1)	
>80	6 (26.1)	60 (37.7)	190 (36.3)	
Female sex, *N* (%)	17 (73.9)	80 (50.3)	288 (55.1)	0.1
Sample source (*N*, %)				
Hospital	19 (82.6)	131 (82.4)	453 (86.6)	0.39
General practice	4 (17.4)	28 (17.6)	70 (13.4)	
Median Elixhauser score (% with data available)	6.70 (56.5)	6.75 (57.2)	Data not available	0.95
Descriptives				
Hospital-onset episodes *N*/total with data available (%)	11/22 (50)	73/141 (51.8)	244/446 (52.3)	0.95
Toxin EIA positive^a^, *N* (%)	14/21 (63.6)	43/98 (43.9)	132/319 (41.3)	0.08
Speciality, *N* (%)				
Medicine	10 (43.5)	83 (52.2)	259 (49.5)	0.69
Surgery	5 (21.7)	23 (14.5)	95 (18.2)	
General practice	4 (17.3)	28 (17.6)	70 (13.4)	
Other	4 (17.3)	25 (15.6)	99 (18.9)	
Biomarkers, median (range)				
CRP (mg/L)	173 (36–319)	44 (1–358)	99 (1–364)	<0.008
Leukocytes (x10^9^/L)	15.0 (6.5–22)	11.2 (2.5–30.3)	11.0 (0.3–51.4)	0.22
Albumin (g/L)	17.5 (11–24)	23 (12–36)	23 (11–48)	0.07
Creatinine (μmol/L)	98.5 (39–229)	80 (38–225)	68 (30–660)	0.36
Outcome				
Length of hospital stay (days) in 30-day survivors, mean (±SD)	15.8 (±20.8)	20.2 (±21.9)	22.7 (±29.1)	0.74
Death within 30 days of sampling, *N*/total with data available (%)	7/17 (41.2)	23/105 (21.9)	60/341 (17.5)	0.02

CRP, C-reactive protein; EIA, enzyme immunoassay; SD, standard deviation.

Data were compared between episodes caused by different ribotypes: RT220, RT002 with RT015, and all other ribotypes.

a Toxin EIA testing undertaken from July 2012.

b Chi-squared test for categorical variables.

**Table II:** Multi-variable Cox regression analysis for 30-day all-cause mortality (*N*=462^a^)

Variables	Unadjusted hazard ratio (95% CI)	*P*-value	Adjusted hazard ratio (95% CI)	*P*-value
Ribotype				
All others	Reference		Reference	
RT002/015	1.28 (0.79–2.08)	0.31	1.20 (0.74–1.94)	0.467
RT220	2.93 (1.34–6.42)	0.007	2.46 (1.06–5.74)	0.037
Age	1.04 (1.02–1.05)	<0.001	1.04 (1.02–1.05)	<0.001
Sex				
Male	Reference		Reference	
Female	0.93 (0.61–1.41)	0.73	0.83 (0.54–1.26)	0.375

CI, confidence interval.

a Thirty-day all-cause mortality data as listed in Table I; demographic data missing for one episode in RT002/RT015 group.

Modified Elixhauser comorbidity scores were available for 57% of episodes with RT220 (13/23) and RT002/RT015 (91/159); there was no significant difference in comorbidity scores identified between episodes associated with RT220 or RT002/RT015 (*P*=0.95) (Table I). RT220 was associated with increased likelihood for toxin EIA detection; however, this was not significant in comparison with RT002/RT015 or all other ribotypes (*P*=0.08). Biomarker results such as CRP, total leukocyte count, albumin and creatinine were available for 441 of 705 (62.6%), 446 of 705 (63.2%), 362 of 705 (51.3%) and 483 of 705 (68.5%) episodes, respectively (Table I and Figure S3, see online supplementary material). RT220 was associated with an elevated median CRP of 173 mg/L (*P*=0.008) and a trend towards reduced albumin (*P*=0.07) compared with RT002/RT015 and all other ribotypes at the time of stool sampling, although differences in creatinine levels (*P*=0.36) and leukocyte count (*P*=0.22) were not apparent (Figure S3, see online supplementary material). Despite an association with increased mortality, a difference in length of hospital stay in 30-day survivors was not associated with RT220 (Table I).

Toxin EIA detection across all ribotypes was associated with elevated CRP and reduced albumin compared with toxin-negative samples: median CRP 126 mg/L (toxin positive) vs 58.5 mg/L (toxin negative, *P*=0.001); and median albumin 22 g/L (toxin positive) vs 25 g/L (toxin negative, *P*=0.003). Toxin production was not associated with differences in leukocyte count or creatinine: median leukocyte count 11.5 vs 11.15 x10^9^/L (*P*=0.28); and median creatinine 76 vs 66 μmol/L (*P*=0.3). Thirty-day mortality (where available) was 24% (31/129) in episodes associated with toxin-positive samples, compared with 17.1% (31/181) in episodes associated with toxin-negative samples (*P*=0.12).

Thirty-seven (5.2%) patients had a recurrent *C. difficile* episode over the study period; 32 patients with two episodes, four patients with three episodes and one patient with four episodes. Of the 32 patients with a single recurrence, 16 had the same ribotype, suggesting a relapse, while 11 patients had a re-infection with a different ribotype; for five patients, culturing *C. difficile* failed. The median recurrence time was 58 days for relapse, compared with 121 days in those re-presenting with a different ribotype (i.e. new infection) (*P*<0.05). Thirteen ribotypes were responsible for relapse: RT005 (four patients, 19%); RT078 (three patients, 14.2%); RT002, RT015 and RT023 with two patients; and RT011, RT012, RT014, RT020, RT076, RT220, RT263 and RT293 with one patient each. RT220 was responsible for three *C. difficile* episodes in one patient. These three episodes occurred over a six-month period; in all, toxin was detected by EIA. Two of the three episodes occurred in the community; however, laboratory biomarkers were only taken during hospital admission. This patient survived.

Two hospital wards accounted for two RT220 cases each; in one ward, there was a gap of 18 days between cases, and in the other ward, the gap between cases was >365 days.

## Discussion

To the authors' knowledge, this is the first large-scale study to report *C. difficile* RT220 as a ribotype associated with increased 30-day mortality and enhanced acute phase response compared with other dominant ribotypes. Importantly, the previously identified severity-associated RT027 was not prevalent; in this study, the most frequently identified ribotypes were RT002 and RT015.

A strength of this study is the inclusion of all clinically diagnosed *C. difficile* episodes in the two-year study period. Furthermore, as the laboratory serves a population of over 2 million people under the care of primary and secondary care providers across a wide area of West London, the data represent an inclusive and substantial urban sample.

The possibility that the association of severity with RT220 might be the result of confounding factors (e.g. increased age) was considered; however, this was not detected. Comorbidity data were not available for many of the patients, but modified Elixhauser scores did not differ significantly between the groups. Review of primary ICD-10 diagnoses among RT220 patients did not reveal an alternative explanation for the high mortality rate, such as specific malignancy. The possibility that RT220 isolates could be associated with an outbreak was considered; however, RT220 isolates were identified over the entire study period, and from multiple locations and specialities, including some samples submitted from the community. Genome sequencing of the RT220 isolates did not demonstrate relatedness between isolates, confirming that these isolates were not from an outbreak, however analysis is still in progress. RT220 demonstrated a trend towards detectable toxin EIA production, consistent with other ribotypes reported to be linked to severe disease [4].

The dominance of RT002 and RT015 in clinical disease was striking, although this is not fully understood. The predominance of RT002 has been reported elsewhere in the UK, Europe and Asia [1], [21], and may reflect higher sporulation frequency in RT002, as seen in epidemic strains of RT027 [22]. Importantly, increased morbidity or mortality was not seen in cases with RT002 in the present study. Regression of RT027 as a leading disease-associated ribotype may be relevant to expansion of new ribotype lineages. Surveillance data from elsewhere in the UK and Europe have yet to identify RT220 among commonly isolated ribotypes [1], [21], [23]; however, in the UK as a whole, RT220 has a prevalence of just over 2% [1], comparable to the prevalence of 3.3% found in this study.

The total recurrence rate in this study (relapse or re-infection) was just 37 of 705 (5.2%), which is lower than a previously reported rate of 20% [24], albeit among exclusively toxin EIA-positive cases. Among the toxin EIA-positive cohort from the present study, 21 of 196 (10.7%) had a recurrent infection. RT005 was most commonly associated with recurrent *C. difficile* in the adult population. In support of previously published work [17], the present study shows that relapse (with the same ribotype) is most likely to occur within the first two months after initial infection, but relapses of symptoms at four months are more likely to be with a different ribotype.

Biomarkers such as CRP and leukocyte count have also been closely associated with toxin production and adverse outcome [24]. The present study found a strong association between high leukocyte count and lower albumin in toxin EIA-positive episodes compared with toxin EIA-negative episodes. In this study, RT220 demonstrated toxin production more frequently than other ribotypes, although this was not significant. Despite these findings, RT220 was not associated with increased length of hospital stay (excluding the patients that died within 30 days); however, missing data and low study patient numbers with this ribotype may have affected the ability to detect such differences. The possibility that the present observations were linked to toxin production, which was not tested before April 2012, was considered. Limiting analysis to toxin EIA-positive isolates alone, mortality was 10.9% (19/175) among cases with non-RT220 toxin-producing isolates but 21% (3/14) among the small number of patients with RT220 toxin-producing isolates (*P*=0.208).

Associations between *C. difficile* ribotype and disease recurrence or severity have been sought by several investigators with variable findings [5], [6], [12], [25], [26]. RT220 comprises, among others, one part of the *C. difficile* multi-locus sequence type (ST) 2 lineage that also includes RT020, RT014 and RT076 [27]. Mortality associated with these ribotypes was 20% (5/25), 17.6% (6/34) and 12.5% (3/10), respectively, where data were available. ST2 has been associated with an increased inflammatory response as well as mucosal disruption in animal models [28]. Ribotype banding patterns are very similar between RT020, RT014 and RT220, and the signal observed for RT220 in the present study may be a surrogate marker of ST2 severity. The most common ribotype within ST2, RT014, has been reported to be associated with increased mortality [5]; however, this association was not detected in the present study. Preliminary genomic analysis indicates that all of the RT220 isolates analysed possess the genes for toxin A (*tcdA*) and toxin B (*tcdB*), but not the binary toxin CDT locus.

Overall mortality at 30 days was 19% in this study, with mortality rates of 17.1% among toxin-negative cases and 23% among toxin-positive cases. Although these data are from 2011–2013, prior to enhanced national guidance regarding *C. difficile* management, the data are consistent with nationally reported mortality over that period [29].

This study was limited by the number of samples that were acquired during the study period, and the fact that mortality data were only available for those samples with complete case identification data. Comorbidity and biomarker data were only available for those admitted to the university hospital. Despite the small sample of patients with RT220, a power of approximately 60% was used to explore a difference in mortality between RT220 and all other ribotypes. The authors were unable to examine individual case notes, so data on treatment given during the episode and prior antibiotic or healthcare exposures were not collected, and the authors were unable to confirm specific causes of mortality among the patient cohort. Comorbidities of those with infections caused by RT002, RT015 and RT220 were examined, where available; modified Elixhauser scores did not reveal any significant differences between the groups that could explain the high mortality rates. The recurrence of RT220 in one female patient may account for the predominance of RT220 episodes in females observed in this study. This recurrence did not lead to death, and biomarkers were only taken on one occasion so did not skew mortality or biomarker parameters. Antimicrobial susceptibilities of *C. difficile* were not examined in this study, although antimicrobial resistance has been implicated in the previous emergence of RT027 [30]. In the small number of isolates tested, RT220 was sensitive to both metronidazole and vancomycin; these antibiotics would have been standard treatment for *C. difficile* cases during the study period.

RT220 is among the leading 10 ribotypes causing infections in the study region and has persisted as a cause of symptomatic disease despite improvements in infection control practices and stewardship. Indeed, in 2018, RT220 accounted for 3.3% (9/268) of *C. difficile* cases in the study region, although it is not known if there is an ongoing association with severity. Whether genomic elements in RT220 confer enhanced pathogenicity or toxicity is unclear, and this is the subject of ongoing research. The identification of RT220 as a ribotype associated with more severe disease and its persistence as one of the more common ribotypes isolated in the study region indicates that ongoing prospective surveillance remains essential.
