# Supplementary material for: Two-year analysis of Clostridium difficile ribotypes associated with increased severity
Source: J Hosp Infect. 2019 Dec;103(4):388–94. doi: 10.1016/j.jhin.2019.06.003 (PMC6926500; doi:10.1016/j.jhin.2019.06.003)
Supplement: Multimedia component 1 [file mmc1.pdf]

Supplementary Data Two year analysis of *Clostridium difficile* ribotypes associated with increased severity

Table S1

Routine data associated with RT220 episodes (*N*=21 patients; 23 episodes)

	Age range (years)	Sex	Speciality[a]	Toxin EIA result	LoS (days) prior to sample	Death within 30 days	Primary diagnosis (ICD-10)
1	31–40	F	Medical	Negative	0	Unk	Intestinal infection
2	31–40	F	Surgical	Not done	5	Unk	Fractured spine
3	51–60	F	Medical	Positive	4	No	Intestinal infection
4	61–70	F	Gynae	Negative	5	No	Ovarian cancer
5	61–70	M	ED	Positive	0	No	Unk
6	61–70	F	Renal	Negative	1	Unk	Pneumonia
7	61–70	M	Surgical	Positive	6	Unk	Hepatobiliary cancer
8	61–70	F	Medical	Not done	0	No	Unk
9	71–80	F	ED	Positive	0	No	Unk
9	71–80	F	Medical	Positive	10	No	Depressive disorder
9	71–80	F	GP	Positive	NA	No	Unk
10	71–80	M	Medical	Negative	0	Yes	Pneumonia
11	71–80	M	GP	Negative	NA	Unk	Unk
12	71–80	F	Medical	Positive	15	Yes	Urosepsis
13	71–80	F	GP	Positive	NA	Yes	Unk
14	71–80	F	Medical	Positive	5	Yes	Pneumonia
15	71–80	F	Surgical	Negative	1	No	Urosepsis
16	81–90	M	Surgical	Positive	25	Unk	Unk
17	81–90	F	Surgical	Positive	5	Yes	Lower gastrointestinal cancer
18	91–100	F	GP	Positive	NA	No	Unk
19	91–100	M	Medical	Positive	19	Yes	Cerebral infarction
20	91–100	F	ICU	Negative	17	Yes	Unk
21	91–100	F	Medical	Positive	1	No	Unk

EIA, enzyme immunoassay; ED, emergency department; GP, general practice; ICU, intensive care unit; F, female; M, male; LoS, length of stay prior to *Clostridium difficile* sample submission; NA, not admitted; Unk, unknown; ICD-10, International Classification of Diseases-10.

Mortality data unknown for six of 21 patients as unable to link to national databases/lack of correct NHS number.

Primary ICD-10 diagnosis unknown for 10 of 23 RT220 episodes if not admitted/not admitted to university hospital group.

Supplementary Data Two year analysis of *Clostridium difficile* ribotypes associated with increased severity

[a]Clinical team submitting sample for *C. difficile* testing at time of diagnosis.

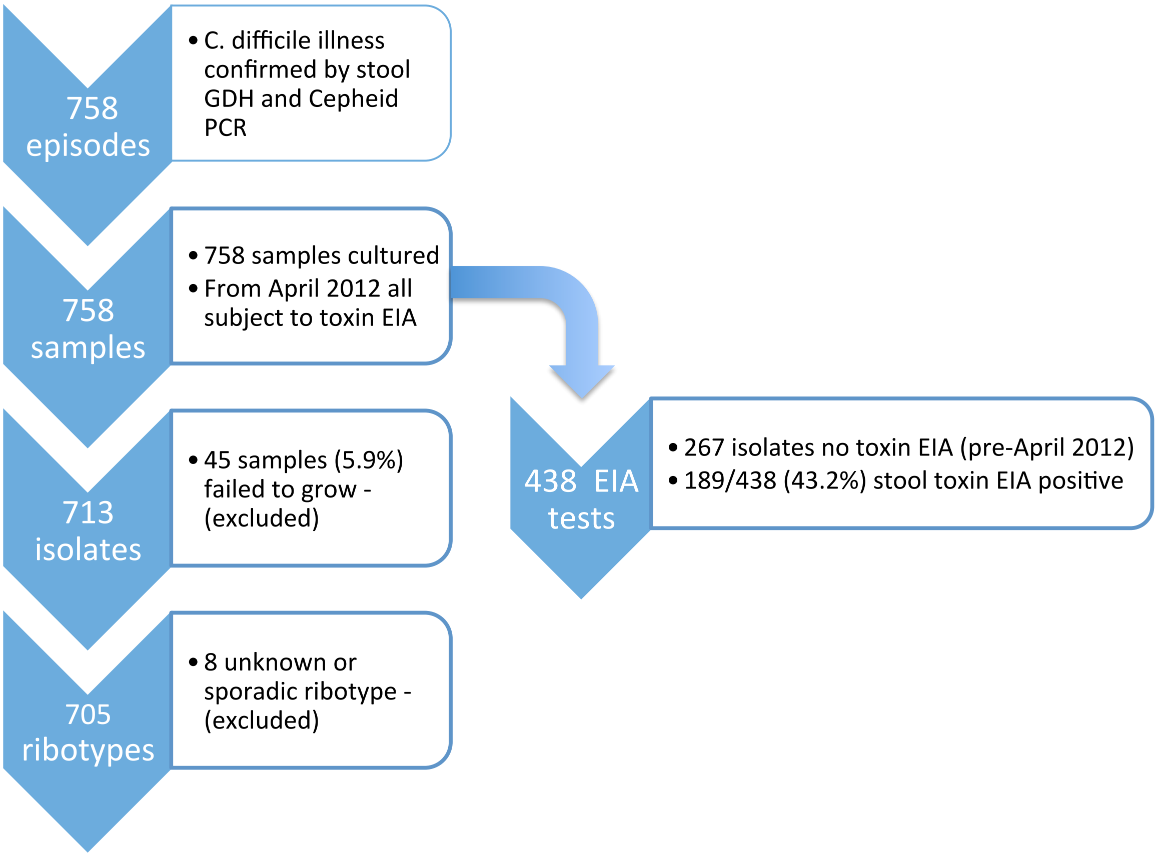

Figure S1. Flow chart outlining inclusion of episodes, isolates and toxin testing. *C. difficile*, *Clostridium difficile*; GDH, glutamate dehydrogenase; PCR, polymerase chain reaction; EIA, enzyme immunoassay.

Supplementary Data Two year analysis of *Clostridium difficile* ribotypes associated with increased severity

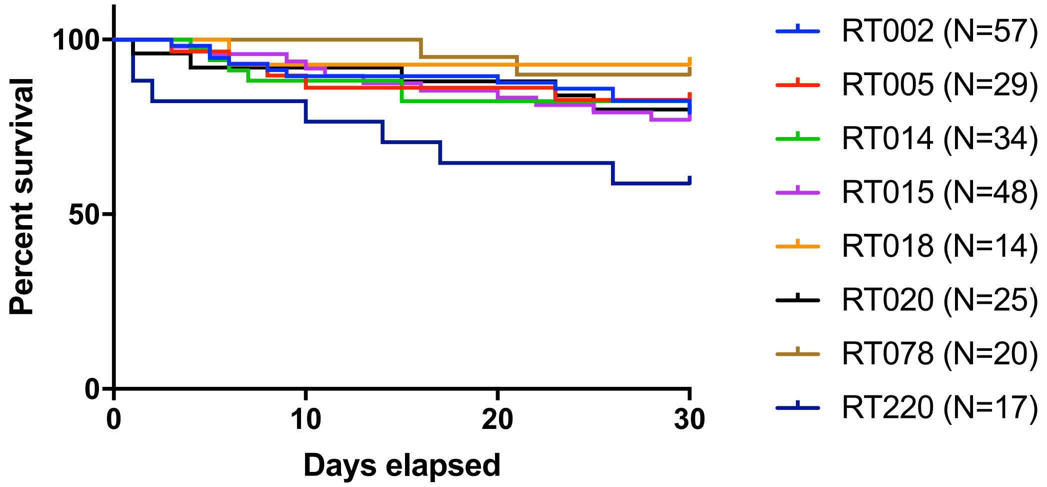

Figure S2. Kaplan–Meier survival plot of 30-day all-cause mortality from date of *Clostridium difficile* sampling by prevalent ribotypes (*N*=number of episodes for which mortality/survival data were available) for ribotypes with prevalence >3%.

Supplementary Data Two year analysis of *Clostridium difficile* ribotypes associated with increased severity

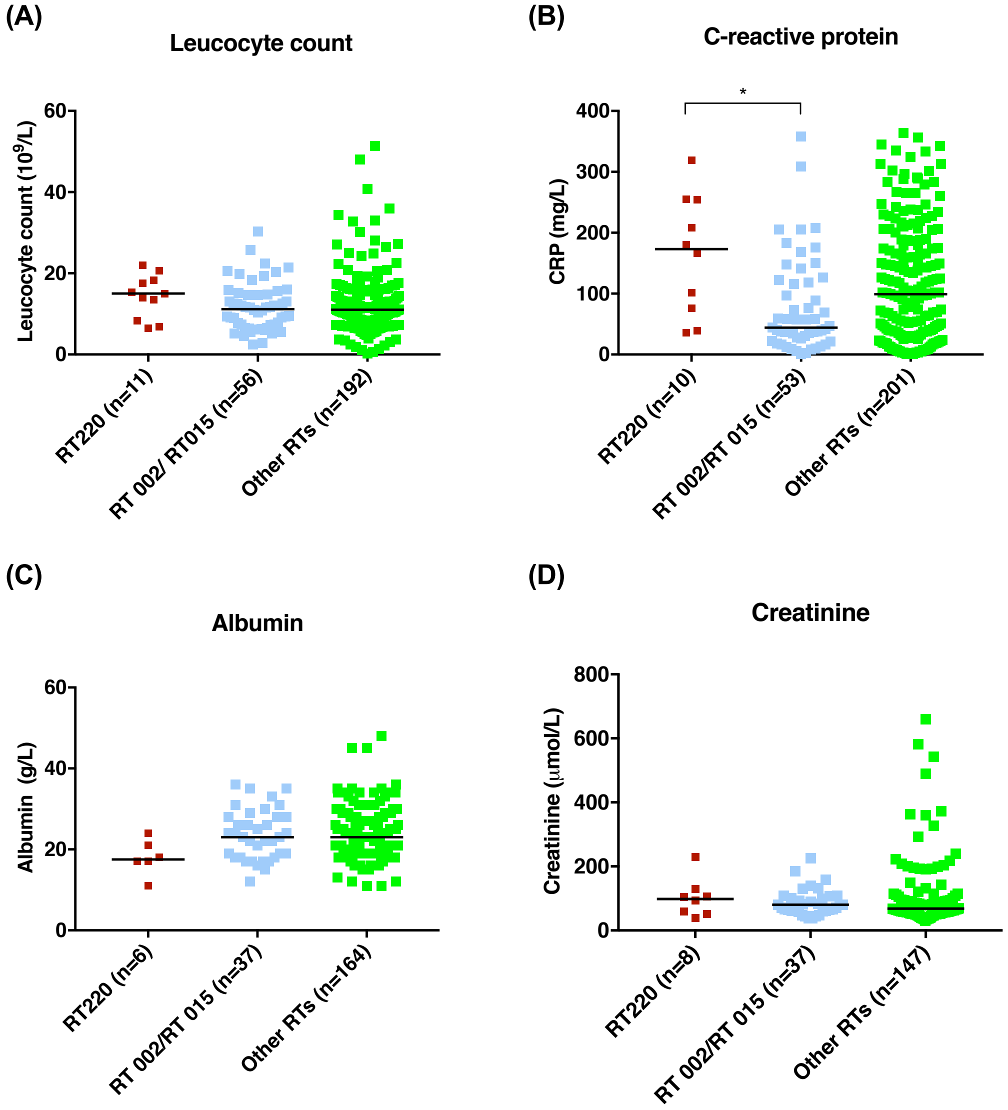

Figure S3. Biomarkers at time of diagnosis of each *Clostridium difficile* episode compared by ribotype. (A) Leukocyte count recorded within 48 h of sampling (normal range 3.6–11.0 10^9/L); no significant association between RT220 and leukocyte count recorded when compared with other ribotypes (*P*=0.22). (B) C-reactive protein (CRP) recorded within 48 h of sampling (normal range <5 mg/dL); RT220 was associated with an elevated median CRP of 173 mg/L (*P*=0.008). (C) Albumin recorded within 48 h of sampling (normal range 35–55 g/L); RT220 showed a trend towards reduced albumin when compared with RT002/RT015 and all other ribotypes (*P*=0.07). (D) Creatinine (excluding renal patients)

recorded within 48 h of sampling (normal range 45–110 µmol/L); no significant associations between RT220 when compared with other ribotypes (*P*=0.36). Each dot represents a unique *C. difficile* episode where biomarkers were available. Horizontal line represents median value. **P*-value <0.05 (Kruskall–Wallis).
